# Evaluation of Loop-Mediated Isothermal Amplification (LAMP) in Urine Samples for the Diagnosis of Imported Schistosomiasis

**DOI:** 10.3390/tropicalmed8120518

**Published:** 2023-12-13

**Authors:** Joaquín Salas-Coronas, María Pilar Luzón-García, Beatriz Crego-Vicente, Manuel Jesús Soriano-Pérez, Begoña Febrer-Sendra, José Vázquez-Villegas, Juan García-Bernalt Diego, Isabel María Cabeza-Barrera, Nerea Castillo-Fernández, Antonio Muro, María Dolores Bargues, Pedro Fernández-Soto

**Affiliations:** 1 Tropical Medicine Unit, Hospital Universitario Poniente, 04700 El Ejido, Almería, Spain; mp.luzon.sspa@juntadeandalucia.es (M.P.L.-G.); manuelj.soriano.sspa@juntadeandalucia.es (M.J.S.-P.); mariai.cabeza.sspa@juntadeandalucia.es (I.M.C.-B.); nerea.castillo.sspa@juntadeandalucia.es (N.C.-F.); 2 Department of Nursing, Physiotherapy and Medicine, Faculty of Health Sciences, University of Almería, 04120 La Cañada, Almería, Spain; 3 Infectious and Tropical Diseases Research Group (e-INTRO), Biomedical Research Institute of Salamanca-Research Centre for Tropical Diseases at the University of Salamanca (IBSAL-CIETUS), Faculty of Pharmacy, University of Salamanca, 37007 Salamanca, Spain; beatrizcregovic@usal.es (B.C.-V.); begofebrer@usal.es (B.F.-S.); juanbernalt95@usal.es (J.G.-B.D.); ama@usal.es (A.M.); 4 Tropical Medicine Unit, Distrito Poniente de Almería, 04700 El Ejido, Almería, Spain; joseb.vazquez.sspa@juntadeandalucia.es; 5 Department of Parasitology, Faculty of Pharmacy, University of Valencia, 46100 Burjassot, Valencia, Spain; m.d.bargues@uv.es; 6 CIBER de Enfermedades Infecciosas, Instituto de Salud Carlos III, 28029 Madrid, Spain

**Keywords:** schistosomiasis, diagnosis, LAMP, immunochromatographic rapid test, microscopy, latent class analysis

## Abstract

Migratory flows and international travel are triggering an increase in imported cases of schistosomiasis in non-endemic countries. The present study aims to evaluate the effectiveness of the LAMP technique on patients’ urine samples for the diagnosis of imported schistosomiasis in a non-endemic area in comparison to a commercial immunochromatographic test and microscopic examination of feces and urine. A prospective observational study was conducted in sub-Saharan migrants attending the Tropical Medicine Unit, Almería, Spain. For schistosomiasis diagnosis, serum samples were tested using an immunochromatographic test (Schistosoma ICT IgG-IgM). Stool and urine samples were examined by microcopy. Urine samples were evaluated by combining three LAMP assays for the specific detection of *Schistosoma mansoni*, *S. haematobium*, and for the genus *Schistosoma*. To evaluate the diagnostic accuracy, a latent class analysis (LCA) was performed. In total, 115 patients were included (92.2% male; median age: 28.3 years). Of these, 21 patients (18.3%) were diagnosed with schistosomiasis confirmed by microscopy, with *S. haematobium* being the most frequent species identified (18/115; 15.7%). The Schistosoma ICT IgG-IgM test result was 100% positive and Schistosoma-LAMP was 61.9% positive, reaching as high as 72.2% for *S. haematobium*. The sensitivity and specificity estimated by LCA, respectively, were: 92% and 76% for Schistosoma ICT IgG-IgM, 68% and 44% for Schistosoma-LAMP, and 46% and 97% for microscopy. In conclusion, the Schistosoma-LAMP technique presented a higher sensitivity than microscopy for the diagnosis of imported urinary schistosomiasis, which could improve the diagnosis of active infection, both in referral centers and in centers with limited experience or scarce resources and infrastructure.

## 1. Introduction

Schistosomiasis is an acute and chronic disease caused by several trematode flatworms (blood flukes) of the genus *Schistosoma*. Currently, 78 countries maintain transmission of the disease, causing a global infection burden of 230 million people, with up to 800 million at risk. Over 90% of them live in sub-Saharan Africa, under poverty or extreme poverty conditions [1,2]. Schistosomiasis is considered one of the most devastating parasitic diseases, and it is one of the neglected tropical diseases (NTDs) [3]. Migratory flows and international travel are triggering an increment in imported cases in non-endemic countries [4,5]. Moreover, population movements, combined with the effects of climate change, have caused autochthonous transmission of the disease in non-tropical regions, such as France [6] and Spain [7].

The availability of highly sensitive diagnostic tests is crucial for the diagnosis of symptomatic cases of schistosomiasis, for mass screening of people at risk, and for the evaluation of eradication programs carried out in endemic regions [8]. To date, there is no reference diagnosis for schistosomiasis. Direct microscopic observation of urine, feces, or tissues shows a low sensitivity, particularly for intestinal schistosomiasis in adults or long-term residents outside endemic areas [2,9]. On the other hand, a number of serological assays detecting antibodies against *Schistosoma* spp. are commercially available and they are recommended by the European Centre for Disease Prevention and Control (ECDC) for screening for schistosomiasis in migrants who have been living in Europe for less than 5 years [10]. However, antibody detection has several limitations, including variable sensitivity, inability to distinguish past and active infections, lack of utility for post-treatment monitoring, and poor performance in the early stages of acute infections [9,11]. Antigenic tests, such as the circulating cathodic antigen (CCA) in urine, also suffer from important limitations for the diagnosis of *Schistosoma* species other than *S. mansoni* and for the diagnosis of patients in non-endemic regions [9,12]. Finally, molecular techniques, in particular PCR-based methods, have also been used for the diagnosis of human schistosomiasis [13], being particularly valuable for the simultaneous detection and identification of *Schistosoma* species [14]. Although very sensitive and accurate, complex PCR-based methods for schistosomiasis are expensive and require specialized personnel and equipment, and are, therefore, not useful for diagnosis under field conditions and are usually only available in reference laboratories [15].

In this context, isothermal nucleic acid amplification tests, in particular the loop-mediated isothermal amplification (LAMP) technology, could be a good alternative because they have several relevant advantages over most PCR-based methods. LAMP is a powerful nucleic acid amplification technique that combines simplicity, elevated sensitivity, and specificity in DNA detection [16]. LAMP technology has all the characteristics required of a high-efficiency diagnostic assay along with simple operation for use in the clinical diagnosis of infectious diseases, including point-of-care (POC) testing under field conditions in developing countries [17,18].

A number of LAMP assays have already been developed and successfully used for the specific detection of the three main species causing human schistosomiasis (*Schistosoma haematobium*, *S. mansoni,* and *S. japonicum*) and have been applied to the diagnosis and evaluation of the efficacy of chemotherapy, both in animal models and human patients. Previous studies by our group have developed and successfully evaluated LAMP assays in the clinical determination of *S. mansoni* in both stool and urine samples, as well as *S. haematobium* in urine samples. In addition, we have described a genus-specific LAMP assay for simultaneous detection of the most important schistosome species affecting humans, including *S. haematobium, S. mansoni,* and *S. intercalatum* [19], which has also been evaluated to detect DNA from *S. haematobium-S. bovis* hybrids [20], but to date has not yet been used with clinical samples.

Thus, the main purpose of this study was to evaluate, the performance of the LAMP technique on patients’ urine samples for the diagnosis of imported schistosomiasis in a non-endemic area.

## 2. Materials and Methods

### 2.1. Study Design and Study Population

A prospective observational study comparing diagnostic tests for the detection and screening of schistosomiasis was carried out in sub-Saharan migrant patients who attended the Tropical Medicine Unit (TMU) of the Hospital Universitario Poniente (El Ejido, Almería, Spain) from January 2020 to June 2021. The Poniente area is an administrative area located in southeast Spain, holding a population close to 300,000 inhabitants with a migrant share of 21%, many of them coming from sub-Saharan countries to work in horticultural greenhouses. Patients included were sub-Saharan migrants older than 14 years of age who agreed to participate in the study and signed the informed consent form. Patients with HIV infection were excluded.

### 2.2. Definitions and Collected Data

A screening protocol for infectious diseases was systematically applied to all migrant patients referred to the TMU. For sub-Saharan migrant patients, the screening protocol comprised medical history, epidemiological data, complete physical examination, and several additional tests: blood count, liver and renal function tests, syphilis, HIV, HBV and HCV serologies, tuberculin skin test and search for parasites in stool (three concentrated stool samples) and urine (one concentrated urine sample [10 mL]), *Strongyloides* (ELISA DRG^®^ Strongyloides IgG. Springfield, Geneseo, IL, USA) and *Schistosoma* serological tests, and Knott and/or saponin tests for microfilariae. Chest and abdominal X-rays were routinely performed too. If any other specific disease was suspected (e.g., onchocerciasis, malaria, etc.), further proper diagnostic procedures were performed. Diagnosis of strongyloidiasis was considered either when larvae were isolated from stool samples or when serology was positive.

Diagnosis of schistosomiasis was confirmed when *Schistosoma* spp. eggs were microscopically detected in urine and/or feces. Patients with confirmed schistosomiasis and those with a serology- and/or Schistosoma-LAMP-positive result were treated with praziquantel at the usual doses (40 mg/kg, 1 day), completing the study with abdominal and bladder ultrasound in cases of confirmed schistosomiasis or when they presented genitourinary symptoms.

### 2.3. Microscopy

Urine microscopy: The urine samples were collected between 9 a.m. and 12 a.m. and processed on the day of the sample collection. After centrifugation and concentration, each urine sample was placed on a labeled slide and examined under a microscope (100×) for *Schistosoma* eggs. Aliquots of urine samples were reserved and stored at −80 °C until shipment to the laboratory at IBSAL-CIETUS (Salamanca, Spain) for further DNA extraction for molecular analyses.

Stool microscopy: Three stool samples per patient were collected in formol on alternate days. Each sample was submitted to formol-ether concentration (Ritchie method) and 3 slides were examined at the microscope (100×).

### 2.4. Schistosoma ICT IgG-IgM Testing

Serum samples were tested for antibodies against *Schistosoma* spp. using the commercial rapid immunochromatographic test Schistosoma ICT IgG-IgM (LDBIO Diagnostics, Lyon, France) according to the manufacturer’s instructions. Briefly, 30 μL of each serum sample were added to a cassette, followed by 3 drops of the supplied eluent in the kit. The result was read after 20–30 min as positive or negative depending on whether or not a colored band appeared.

### 2.5. LAMP Testing

Aliquots of 2 mL of patients’ frozen urine samples were used for DNA extraction for molecular analysis by using the NZY Tissue gDNA Isolation kit (NZYtech, Lda., Lisbon, Portugal) following the manufacturer’s instructions. Subsequently, 2 μL of purified DNA thus obtained were added as template for LAMP amplifications. LAMP technique was performed using the combination of three different LAMP assays (hereafter, Schistosoma-LAMP assays) previously described elsewhere by our research group (IBSAL-CIETUS) for the specific detection of *Schistosoma mansoni* (*Sm*-LAMP), the specific detection of *S. haematobium* (*Sh*-LAMP), and an assay for the genus *Schistosoma* (*Schisto*-LAMP) [19].

All the Schistosoma-LAMP assays were performed similarly. In brief, LAMP reaction mixtures (15 μL) contained 1.6 M FIP/BIP primers, 0.2 μM F3/B3 primers, 0.4 μM LF/LB primers (if applicable), 1.4 mM of each dNTP, (Bioron, GmBH, Römerberg, Germany), 6 mM MgSO_4_, and 1× isothermal amplification buffer (20 mM Tris-HCl (pH 8.8), 50 mM KCl, 10 mM (NH_4_)_2_SO_4_, 2 mM MgSO_4_, 0.1% Tween20) for Bst 2.0 Warm Start (WS) (0.32 U/μL) (New England Biolabs Ltd., Ipswich, MA, USA) and 2 µL of template DNA. Reactions were performed in 0.5 mL micro-centrifuge tubes by incubation in a heating block at 65 °C for 60 min followed at 80 °C for 5–10 min to stop the reaction. The LAMP amplification results were visually inspected by adding 1 μL of 1:10 diluted 10,000× concentration fluorescent dye SYBR Green I (Invitrogen, Waltham, MA, USA) in each reaction tube post-amplification. Green fluorescence was observed in LAMP-positive reactions and original orange in LAMP-negative reactions. Positive (DNA from *S. mansoni*, *S. haematobium* or both) and negative controls (purified water instead DNA template) were included in all trials. The tubes were briefly centrifuged and carefully opened before adding the dye to avoid possible cross-contamination with amplified products.

### 2.6. Statistical Analysis

For descriptive statistical analysis, quantitative variables were expressed as means ± standard deviations (SD) and qualitative variables as frequencies and percentages. To evaluate Schistosoma-LAMP assays diagnostic capacity compared to direct microscopic observation and immunological test, a latent class analysis (LCA) was performed. LCA combines the results of multiple diagnostic tests through a probabilistic model to obtain estimates of disease prevalence and diagnostic test accuracy in situations where there is no single, accurate reference standard [21]. LCA enables the creation of a hypothetical standard (as it is not known whether patients are infected or not) and determines the test diagnostic capacity based on that standard. The main limitations of this approach are the dependency on the prior’s distributions, given that its choice can influence the results; the computational intensity and complexity; and the independence assumption between the diagnostic tests.

Initially, the apparent prevalence (function of the positives in the evaluated test) and the real prevalence (function of the positives by direct observation, considered “real positives”), sensitivity and specificity (95% confidence interval [CI95%]), positive predictive value (PPV), and negative predictive value (NPV) were obtained. Diagnostic accuracy of each test was also estimated, defined as the true positives fraction in relation to the reference diagnosis.

Then, a LCA was performed, assuming that the number of latent classes was 2 (presence/absence of infection). A Bayesian approach was taken, firstly by defining the frequency of the different tests combination and, secondly, determining the initial conditions of the model. In this case, to properly handle the different scenarios, initial conditions included assumptions of low (10%), medium (50%), and high (90%) prevalence, sensitivity, and specificity. Lastly, *a priori* prevalence, sensitivity, and specificity, informative distributions of each test were obtained, for which estimations of α and β parameters were obtained from the literature on the different tests and the confidence in the truthfulness of the indicated values. Thus, a Bayesian LCA analysis with *a priori* informative distributions was performed. To obtain the parameters and beta distribution, the following values were selected: (1) Prevalence: considering 20% as the most plausible value, with a CI95%, real prevalence was considered to be over 8%. (2) Sensitivity: for direct observation, considering the most plausible value 50% [2] and a CI95%, real sensitivity was considered to be over 40%. For immunological test, the most plausible value considered was 95% [9], CI95%, real sensitivity was estimated at 90%. For Schistosoma-LAMP, the most plausible value considered was 90% [19], CI95%, real sensitivity was estimated at 70%. (3) Specificity: for direct observation, considering the most plausible value 98% and a CI95%, real sensitivity was considered to be over 40%. For Schistosoma-LAMP and immunological tests, the most plausible value considered was 90% (CI95%), real sensitivity was estimated at 70% [9,19].

Statistical analysis was performed in R (version 4.2.2) using packages R2jags, BayesLCA, and randomLCA to analyze the data; α and β parameters of the Beta distribution were obtained with the package epiR.

## 3. Results

A total of 115 patients were finally included in the study. Most of them were male (92.2%) and the mean age was 28.3 years. The mean period of residence in Europe was 30.16 months. The most frequent countries of origin were Senegal (*n* = 43; 37.4%), Mali (*n* = 39; 33.9%), and Guinea Bissau (*n* = 12; 10.4%). Main socio-demographic data and parasitological findings are shown in Table 1.

For all 115 patients, immunological test and urine microscopy data were obtained; stool microscopy data were available for only 108 patients. In the other 7 patients it was not possible to obtain a stool sample, mostly because the initial sample collection was faulty and they subsequently failed to attend the scheduled review that would have allowed a new sample to be requested. A total of 21 patients (21/115; 18.3%) were diagnosed with schistosomiasis, confirmed by microscopic observation, with *S. haematobium* being the most frequent causative agent identified (18/115; 15.7%), all of them in urine samples. Three patients were diagnosed by stool microscopy with intestinal schistosomiasis: *S. mansoni* was identified in two patients and *S. intercalatum*/*S. guineensis* in one patient from Equatorial Guinea. According to the microscopy analysis, the egg numbers in urine varied from 0.1–56 eggs/10 mL, and a median of 6.9 (95%CI [2.8,10.50]), and in feces from a mean of 39.23 (SD 56.09).

The systematic screening protocol for searching parasites detected other helminths, including *Strongyloides stercoralis* (*n* = 12; 10.4%), hookworms (*n* = 3; 2.6%), *Mansonella perstans* (*n* = 1; 0.9%), and *Hymenolepis nana* (*n* = 1; 0.9%). Only one patient with confirmed schistosomiasis (*S. intercalatum*/*S. guineenses*) was coinfected with *Strongyloides stercoralis.* One patient was coinfected with *S. stercoralis* and hookworm.

The results of the Schistosoma ICT IgG-IgM test and Schistosoma-LAMP assays are shown in Table 2. In patients with microscopy-confirmed schistosomiasis, the Schistosoma ICT IgG-IgM was 100% positive and Schistosoma-LAMP was 61.9% positive, with differences among species. In patients without microscopy-confirmed schistosomiasis, the Schistosoma ICT IgG-IgM test was positive in 48.9% and LAMP in 63.8% of cases.

The sensitivity, specificity, PPV, and NPV values estimated by LCA are summarized in Table 3. Overall, the highest sensitivity resulted for Schistosoma ICT IgG-IgM test, the sensitivity of Schistosoma-LAMP was superior to that of microscopy, while its specificity was the lowest of the three.

**Figure 1:**
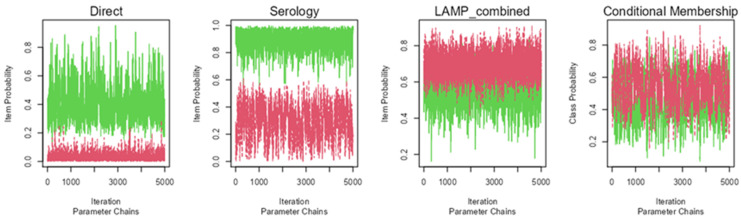
Plots of the convergence of the Markov chains for the informative Bayesian model, with the number of iterations of the algorithm and class probability as X and Y axes, respectively, and the estimated classes represented with red and green colors (presence or absence of infection), with colors inverted for Schistosoma-LAMP assay. Quality of convergence represented by lines showing no trend, low height, and located around 0 and 1 probabilities.

**Figure 2:**
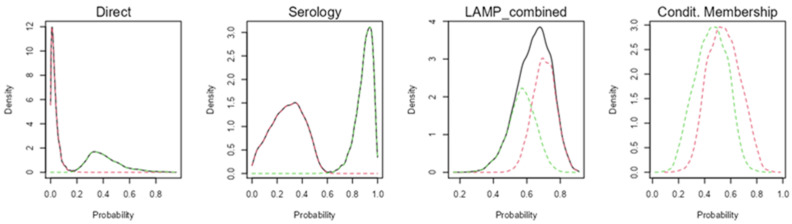
Class probability density plots on the basis of the diagnostic tests, where the estimated classes are represented with red and green colors (presence or absence of infection), with color pattern inverted for Schistosoma-LAMP assay. The higher the density around 0 (for class 1) or 1 (for class 2), the greater the discrimination between classes.

The Bayesian model showed an appropriate convergence, as none of the statistical models of Rubin and Gelman reached values over 1.01 (Figure 1). Probability density graphs for the classes estimated by the models are shown in Figure 2 in order to identify how classes are discriminated.

## 4. Discussion

In Western travel clinics and hospital settings, where schistosomiasis is found as a generally mild imported disease, early diagnosis is an essential priority. This study presents the evaluation of a simple colorimetric LAMP technique in urine samples for the diagnosis of imported schistosomiasis, comparing it with direct microscopic observation and a highly sensitive serological technique. Based on the results, the LAMP technique presents a higher sensitivity than microscopy for the diagnosis of urinary schistosomiasis, which could improve the diagnosis of active infection both in specialized centers and in centers with little experience or scarce resources and infrastructure.

According to data from reference centers in Europe, the prevalence of schistosomiasis identified by microscopy in a series of patients from schistosomiasis-endemic regions ranges from 9% to 17%. However, many of these patients had positive serology with negative microscopy, indicating that schistosomiasis could be misdiagnosed [22,23]. Bearing in mind that the reference centers have personnel with expertise in the microscopic diagnosis of schistosomiasis, the number of potential cases diagnosed in primary care, with technical staff less experienced in the management of imported diseases, is likely to be significantly lower; this could result in an increase in the number of persons with severe complications of the disease.

Currently, serological techniques are recommended for screening of schistosomiasis because of their higher sensitivity [10], being especially useful in individuals with light infections with a negative parasitological test [24]. However, serological tests do not discriminate between active or past infection [2,9,15,24], so this strategy typically results in the treatment of people who are not really sick. Thus, serological data should be considered with caution, given that an overdiagnosis may occur.

In a retrospective study by Beltrame et al. conducted on African migrants for the screening of human schistosomiasis using different tests, LCA assessment indicated an ICT sensitivity of 96% for the diagnosis of imported schistosomiasis, compared to circulating cathodic antigen (CCA) urine cassette (29%), microscopy (48%), ELISA (76%), and Western blot (94%) [9]. According to these results, authors suggest that a dipstick CCA test in a schistosomiasis non-endemic setting is inadequate for screening purposes and the rapid diagnostic test ICT is a suitable screening tool for schistosomiasis, although a positive result should ideally be confirmed by a second test, such as ELISA and microscopy. Although CCA urine cassette test has been recommended as an appropriate tool for monitoring schistosomiasis control programs in endemic areas [15], its low sensitivity obtained in African migrants is probably related to the fact that the parasitic load in the migrant population is much lower than in the population of schistosomiasis-endemic regions of origin. In this sense, it is important to note that the sensitivity values estimated by LCA in our study for microscopy (46.27%) and Schistosoma ICT IgG-IgM test (92.46%) are in agreement with those reported by Beltrame et al. (2017) [9].

The limitations of serological methods make it a priority to develop molecular diagnostic techniques for schistosomiasis that have high sensitivity, detect active infections, and, ideally, do not require expert personnel and large technical resources to carry out. An easy, rapid, and simple colorimetric LAMP assay could meet these requirements [19]. In addition, the use of urine samples instead of other, more difficult to access samples (such as feces, blood or biopsies) greatly simplifies the process, reducing the logistics required in schistosomiasis-endemic regions with limited resources and also at a reference laboratory. Furthermore, in clinical practice, the use of urine specimens would greatly simplify sampling, as a very significant number of sub-Saharan migrants are reluctant to repeat blood sampling.

There are already numerous well-established applications of LAMP technology in the diagnostic of bacterial, viral, fungal, and parasitic diseases in humans, animals, and plants, being particularly useful as a point-of-care (POC) molecular tool for parasitic diseases in resource-limited regions [25]. Moreover, the World Health Organization (WHO) has also recommended the development of LAMP technology for several NTDs, including schistosomiasis [26]. Different studies have been conducted to evaluate the clinical application of LAMP in the diagnosis of human schistosomiasis [19]. In a previous study of our group, a colorimetric LAMP assay was evaluated under field conditions in Cubal (Angola) to detect *S. haematobium* using both purified DNA and crude urine samples in comparison with microscopy. The overall prevalence by LAMP was significantly higher than microscopy when testing both purified DNA (73.8% vs. 50.6%) and crude urine (63.4% vs. 50.6%) samples, respectively [19]. Bayoumi et al. [27] also evaluated a LAMP test to detect *S. haematobium* DNA in urine samples collected from patients suspected of urogenital schistosomiasis attending an outpatient clinic in Egypt. LAMP resulted in a 100% sensitivity and 63.16% specificity when compared with conventional urine filtration followed by microscopy for eggs detection.

To the best of our knowledge, the present study is the first to use the LAMP technique on urine samples in real clinical practice for the diagnosis of imported schistosomiasis. Here, the overall sensitivity for Schistosoma-LAMP estimated by the Bayesian LCA approach was 68.8%, decreasing slightly to 61.9% when using microscopy as the reference method for diagnosis. However, if only *S. haematobium* infections are considered, the sensitivity of Schistosoma-LAMP was as high as 72.2%. These results seem logical, as for the rest of the schistosome species (although detection of eggs in urine is possible), urine is not the ideal sample for detection and LAMP would have a lower sensitivity and, therefore, less diagnostic utility. Given the biological characteristics of the *Schistosoma* species that cause intestinal schistosomiasis, it also seems logical that the LAMP technique would show a higher sensitivity when performed on stool or blood samples.

In a previous work by our group with patients attending the Hospital Universitario Insular de Las Palmas de Gran Canaria (Spain) as part of public health diagnostic activities, colorimetric S. haematobium-specific LAMP was retrospectively evaluated in 18 urine samples from sub-Saharan migrants with parasitological proven *S. haematobium* infection. The diagnostic parameters for LAMP resulted in a sensitivity of 100% and a specificity of 86.67% [19]. Considering that the referred LAMP method is the same as the one used in this study (specifically, the *Sh*-LAMP) the different sensitivity value in comparison to that obtained here (100% vs. 72%, for species-specific *S. haematobium*) could be due to the fact that the patients included in the present study had an average stay in Europe of more than 2.5 years, whereas the patients included in the study conducted in the Canary Islands were newly arrived patients, so they were expected to have a much higher parasite load. The difference in the specificity values obtained between the two studies (86.67%, in Canary Islands vs. 44.60%, in this study), could be due to the fact that specificity was estimated by the Bayesian LCA approach, considering together possible detection of *S. haematobium*, *S. mansoni*, and *S. intercalatum*/*S.guineensis*, which would considerably decrease the specificity.

A number of molecular techniques for the detection and quantification of schistosome-specific DNA in clinical samples have been described [15,24,28] and have proven to be particularly useful for the diagnosis of acute schistosomiasis, especially in travelers, where there is a need for ultra-sensitive blood-based diagnostic tests that can detect *Schistosoma* infection at an early stage [29]. In a recent study with 376 travelers and African migrants from a non-endemic setting in Germany, different diagnostic tests, including microscopy, serology, POC-CAA urine tests, and a serum in-house PCR were performed and the results were analyzed by LCA [28]. For serum in-house PCR (including serology in the calculation), an LCA analysis assessed a sensitivity and specificity of 94.9% and 98.4%, respectively, thus demonstrating that PCR in serum is a highly reliable diagnostic method for schistosomiasis in travelers and migrants. However, to date, there are no studies assessing the efficacy of urine PCR for the diagnosis of schistosomiasis in the migrant population. Notwithstanding this, PCR-based methods are technically demanding, require well-equipped laboratories, well-trained technicians, and are quite expensive, so PCR tests are limited to reference laboratories. Ideally, to overcome these difficulties, PCR-based methods could be replaced by simpler isothermal methods, such as LAMP assay. Unfortunately, to date, there are still no studies evaluating the diagnostic ability of LAMP versus microscopy and/or serology in a non-schistosomiasis endemic clinical setting to compare with our results from a migrant population.

We are aware of some limitations of our work. On one hand, the prevalence estimates in a Tropical Medicine Unit scenario such as the one in which we conducted this study, may not be representative of what occurs in the general population. On the other hand, the use of a single sample and the small amount used for molecular analysis by LAMP (2 µL of DNA extracted from only 2 mL of total urine volume per patient) might have underestimated the diagnosis. This could be especially relevant considering the low parasite load and, therefore, the minimal presence of free circulating parasite DNA in the urine of our patients. Additionally, frozen storage conditions of urine samples without a preserving solution or repetitive freeze–thaw cycles for different trials could be also the reason for a decrease in sensitivity of DNA detection by LAMP. We described similar complications in obtaining positive results in *S. mansoni* DNA detection in urine samples, not only by LAMP but also by PCR [30]. As we also previously pointed out, because the urine was collected by the patients themselves, molecular analysis can be very susceptible to preanalytical contamination issues. In this respect, it would be interesting to use reagents that stabilize cell-free DNA in urine specimens, which would allow efficient collection, transportation, and storage of samples for subsequent molecular analysis [19]. It is also important to note that processes such as hybridization among *Schistosoma* species, mainly *S. haematobium*-*S. bovis,* could cause the sharing of different ITS (internal transcribed spacer) types [31], which would likely affect the sensitivity of LAMP in detecting genomic DNA from the different hybrid specimens. It would be very interesting to test if LAMP assay could amplify the increasingly surprising hybridizations between schistosomes species that infect humans [32,33], considering that this is an emerging problem both in endemic [34] and non-endemic areas of schistosomiasis [6,7]. It would also be very interesting to evaluate the correlation of the sensitivity of LAMP in urine compared to its application in blood and stool samples, which are much more suitable (particularly stool samples) for the detection of genomic DNA of other schistosome species than *S. haematobium.*

Finally, further studies are needed to determine the potential usefulness of LAMP in the follow-up of patients after treatment. If a positive LAMP result is proven negative after treatment, this could be a very important step towards obtaining an easy and inexpensive molecular test that would assure clinicians of treatment success. It would also allow clinical trials to establish the best treatment regimen for patients with urinary schistosomiasis.

## 5. Conclusions

In conclusion, the Schistosoma-LAMP in urine specimens have proved to be more efficient than microscopy for schistosomiasis diagnosis in a migrant population. The easy to perform colorimetric Schistosoma-LAMP might improve the diagnosis of active urogenital schistosomiasis in referral centers and, in particular, in those centers with limited experience in diagnosis and management of imported or tropical diseases.

## Figures and Tables

**Table 1:** Main socio-demographic data and parasitological findings of the 115 patients included in this study.

Data	Overall
**Socio-demographic data**	
Age (mean, SD)	28.3 (6.89)
Male	106 (92.2%)
Months in Europe (mean, SD)	30.16 (33.67)
Country of origin	
Senegal	43 (37.4%)
Mali	39 (33.9%)
Guinea Bissau	12 (10.4%)
Gambia	7 (6.1%)
Mauritania	4 (3.5%)
Burkina Faso	3 (2.6%)
Guinea-Conakry	2 (1.7%)
Equatorial Guinea	2 (1.7%)
Ghana	2 (1.7%)
Ivory Coast	1 (0.9%)
**Confirmed schistosomiasis**	21 (18.3%)
*Schistosoma haematobium*	18 (15.7%)
*Schistosoma mansoni*	2 (1.7%)
*Schistosoma intercalatum/Schistosoma guineensis*	1 (0.9%)
**Other helminths findings**	
*Strongyloides stercoralis*	12 (10.4%)
Hookworms	3 (2.6%)
*Mansonella perstans*	1 (0.9%)
*Hymenolepis nana*	1 (0.9%)

SD, standard deviation.

**Table 2:** Schistosoma ICT IgG-IgM and Schistosoma-LAMP results for schistosomiasis diagnosis compared to direct microscopic observation.

Direct Microscopic Observation *n* = 115	Schistosoma ICT IgG-IgM *n* = 115	Schistosoma-LAMP *n* = 115
**Confirmed schistosomiasis *n* = 21**	21/21 (100%)	13/21 (61.9%)
*S. haematobium n* = 18	18/18 (100%)	13/18 (72.2%)
*S. mansoni n* = 2	2/2 (100%)	0/2 (0%)
*S. intercalatum*/*S. guineensis n* = 1	1/1 (100%)	0/1 (0%)
**Without confirmed schistosomiasis *n* = 94**	46/94 (48.9%)	62/94 (63.8%)

**Table 3:** Accuracy and predictive values estimated by the Bayesian LCA for microscopy, Schistosoma ICT IgG-IgM, and Schistosoma-LAMP tests.

Test	Sensitivity	Specificity	PPV	NPV
Microscopy	46.27 (36.05, 57.81)	97.33 (94.08, 99.24)	93.56	68.33
Schistosoma ICT IgG-IgM	92.46 (82.80, 98.29)	76.86 (60.77, 93.95)	77.04	92.39
Schistosoma-LAMP	68.85 (55.03, 82.21)	44.60 (32.78, 56.98)	51.01	63.048

In brackets: bootstrap 95% confidence interval (CI95).

## Data Availability

The datasets generated during and/or analyzed during the current study are available from the corresponding author on reasonable request.
